# Removal Performance and Mechanism of Emerging Pollutant Chloroquine Phosphate from Water by Iron and Magnesium Co-Modified Rape Straw Biochar

**DOI:** 10.3390/molecules28083290

**Published:** 2023-04-07

**Authors:** Hongwei Sun, Jinjin He, Yucan Liu, Xianguo Ji, Gang Wang, Xiaoyong Yang, Yanxiang Zhang

**Affiliations:** 1 School of Environmental and Materials Engineering, Yantai University, Yantai 264005, China; 2 School of Civil Engineering, Yantai University, Yantai 264005, China

**Keywords:** biochar, chloroquine phosphate, Fe/Mg, modification, adsorption mechanism

## Abstract

Chloroquine phosphate (CQP) is effective in treating coronavirus disease 2019 (COVID-19); thus, its usage is rapidly increasing, which may pose a potential hazard to the environment and living organisms. However, there are limited findings on the removal of CQP in water. Herein, iron and magnesium co-modified rape straw biochar (Fe/Mg-RSB) was prepared to remove CQP from the aqueous solution. The results showed that Fe and Mg co-modification enhanced the adsorption efficiency of rape straw biochar (RSB) for CQP with the maximum adsorption capacity of 42.93 mg/g (at 308 K), which was about two times higher than that of RSB. The adsorption kinetics and isotherms analysis, as well as the physicochemical characterization analysis, demonstrated that the adsorption of CQP onto Fe/Mg-RSB was caused by the synergistic effect of pore filling, π-π interaction, hydrogen bonding, surface complexation, and electrostatic interaction. In addition, although solution pH and ionic strength affected the adsorption performance of CQP, Fe/Mg-RSB still had a high adsorption capability for CQP. Column adsorption experiments revealed that the Yoon–Nelson model better described the dynamic adsorption behavior of Fe/Mg-RSB. Furthermore, Fe/Mg-RSB had the potential for repeated use. Therefore, Fe and Mg co-modified biochar could be used for the remediation of CQP from contaminated water.

## 1. Introduction

The outbreak of the coronavirus disease 2019 (COVID-19) pandemic, which may cause severe acute respiratory syndrome and aggravate underlying diseases, has led to more than 650 million infections and over 6 million deaths worldwide in the recent three years [1] (WHO, 2023). In the fight against COVID-19, chloroquine phosphate (CQP) is considered an effective medicine for treating COVID-19 [2], resulting in further increasing its production and consumption. As an example, the annual production of CQP in China increased by 20% in 2021 [3]. Despite the therapeutic effect, a high dose of CQP may be harmful to human health. Researchers found that 25 µmol/L of CQP was highly toxic to the human body and could cause death [4]. Moreover, CQP may migrate into the environment due to the difficulty of removing from wastewater [5]. Environmental exposure may disturb the physiological functions of animals and plants and lead to the generation of resistant bacteria [6,7]. Thus, it is urgent to find a simple and efficient method to treat CQP-contaminated water.

To date, an advanced oxidation process has been used for the degradation of CQP in water. For instance, Yi et al. studied the degradation of CQP by the synergistic effect of PDINH/MIL-88A and persulfate [8]. The results showed that CQP (10 mg/L) degradation efficiency was 94.6% in 30 min. Peng et al. investigated the degradation of CQP by activating peroxymonosulfate with SA Co-N-C(30) catalyst and found that the degradation rate of CQP (10 mg/L) reached 97.5% within 30 min [9]. Although the advanced oxidation process is an effective method for removing CQP, the high toxicity of the degradation products and high-cost limit its widespread use [10]. In addition, the bioremediation technique is recommended to remove emerging pollutants [11]. Nevertheless, the removal rate of pollutants is low and resistant bacteria may be generated [11]. The adsorption method has received great attention for removing emerging pollutants due to its high efficiency, low cost, simple operation, and ecological feasibility [12].

The successful utilization of adsorption technology relies on the selection of an appropriate adsorbent [13,14,15]. For example, agar–graphene oxide hydrogel was used as an adsorbent for the removal of CQP and showed a high adsorption capacity (31 mg/g) [16]. Nitrogen-rich conjugated microporous polymers were prepared for the adsorption of CQP and demonstrated an excellent adsorption capacity (334.70 mg/g) [17]. However, the application of the above materials was restricted by the high cost and complex preparation process. Biocahr, a next-generation of carbon material, has attracted great attention in the removal of pharmaceuticals (such as sulfamethoxazole, ciprofloxacin, and tetracycline) because of its low cost, numerous pore structure, and functional groups [18,19,20,21]. Thus, biochar presents a high potential in removing CQP, whereas it is found that the adsorption capacity of raw biochar is poor [22]. Therefore, some modifications to raw biochar physicochemical properties are needed.

The loading of metals is in favor of improving the adsorption capacity of raw biochar through developing mesoporous structure and increasing surface area [23]. Since iron and magnesium have abundant reserves and low toxicity, they are recognized as ideal and effective modifiers [22,24]. Iron-modified biochar is widely used as an adsorbent to remove organic pollutants [21,24]. Magnesium-modified biochar has also been reported to increase the removal of organic pollutants [22]. In addition, iron and magnesium co-modified biochar has been applied for the removal of trace metal(loid)s [25], tetracycline [26], and doxycycline [27] from wastewater. However, the adsorption removal of CQP by iron and magnesium co-modified biochar has not been reported.

This study aims to prepare iron and magnesium co-modified biochar (Fe/Mg-RSB) to remove CQP from an aqueous solution. Especially, Fe/Mg-RSB was synthesized via the impregnation–calcination method by controlling the molar ratio of Fe/Mg. Additionally, multiple characterizations (BET, SEM, FTIR, and XPS) and batch experiments (analysis of adsorption kinetics, isotherms, and pH effects) were performed to investigate the CQP removal mechanism by Fe/Mg-RSB. Furthermore, column experiments were carried out to evaluate the engineering application potential of Fe/Mg-RSB.

## 2. Results and Discussion

### 2.1. Characterization of Prepared Biochars

**Figure 1:**
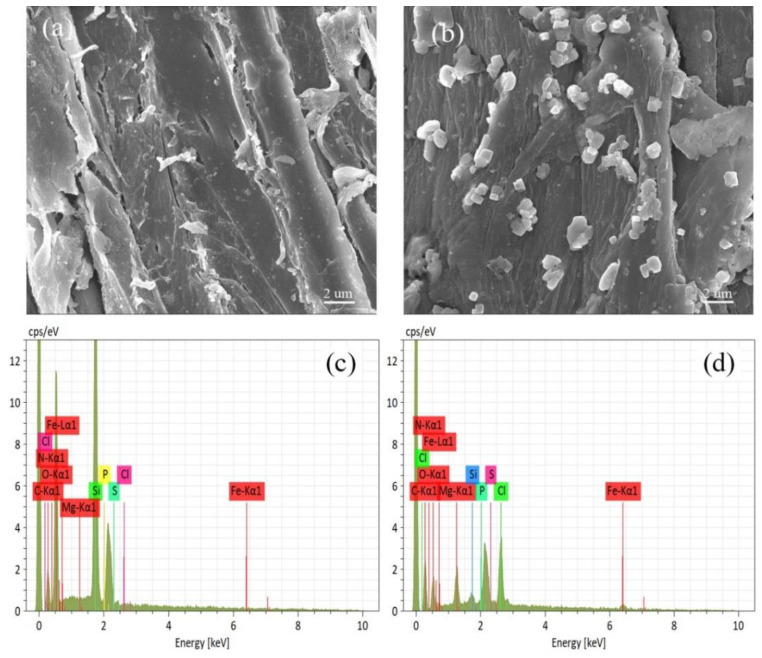
SEM of RSB (**a**) and Fe/Mg-RSB (**b**); EDS of RSB (**c**) and Fe/Mg-RSB (**d**).

The surface structure of RSB and Fe/Mg-RSB were studied by SEM (Figure 1). The surface of RSB was smooth (Figure 1a), while the surface of Fe/Mg-RSB was rough with plenty of particles (Figure 1b), suggesting that the functional modification was successful. The EDS analysis was adopted to further explore the chemical compositions of RSB and Fe/Mg-RSB. The results indicated that RSB was mainly comprised of C and N (Figure 1c). Except for the above two elements, the existence of Fe and Mg was also confirmed on the modified biochar, implying that they were heterogeneously distributed on the RSB surface.

**Figure 2:**
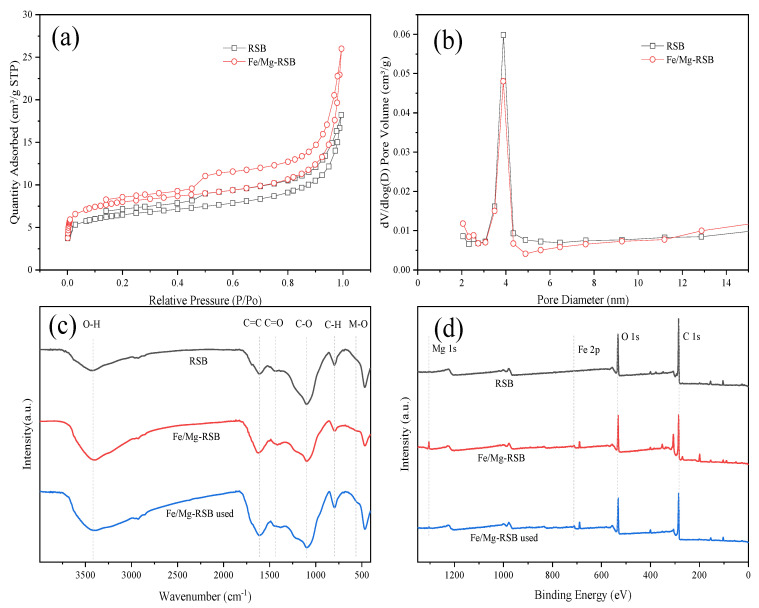
Nitrogen adsorption–desorption isotherms (**a**). Pore size distribution (**b**). FTIR (**c**) and XPS survey (**d**) of biochars.

The nitrogen adsorption–desorption isotherms of biochars are presented in Figure 2a. RSB and Fe/Mg-RSB displayed type IV isotherms with obvious hysteresis loops, suggesting the existence of mesoporous on the biochars [28]. The pore diameter of RSB and Fe/Mg-RSB was mainly around 4 nm based on pore size distribution (Figure 2b). The pore characterization parameters of RSB and Fe/Mg-RSB are shown in Table S1. Compared with RSB, Fe/Mg-RSB displayed a larger surface area (RSB, 20.7053 m^2^/g; Fe/Mg-RSB, 25.3346 m^2^/g) and pore volume (RSB, 0.0189 cm^3^/g; Fe/Mg-RSB, 0.0231 cm^3^/g). The above phenomenon might be due to the following reasons: (1) Fe catalysis could decompose adjacent carbon materials to form low-molecular gases (CH_4_ and CO) and was conducive to pore-forming during the pyrolysis process [29]; (2) The decomposition of MgCl_2_ during the pyrolysis process resulted in the release of HCl and H_2_O and the formation of pore structure [30]; (3) The loaded metal oxides were also contributed to the development of pores [31]. Thus, Fe and Mg co-modification increased the surface area and pore volume, which was beneficial to the adsorption of organic contaminants.

A surface functional group is a key factor influencing the contaminant adsorption property of the biochar [32], which could be characterized by FTIR spectra. The FTIR spectra of biochars were obtained between 4000 and 400 cm^−1^ wavenumbers. As shown in Figure 2c, the peak at 3340−3450 cm^−1^, 1615 cm^−1^, 1440 cm^−1^, 1100 cm^−1^, and 795 cm^−1^ in all biochar samples were respectively ascribed as −OH stretching, aromatic C=C, aromatic C=O, C−O group, and aromatic C−H group. After Fe and Mg co-modification, the intensity of −OH, C=C, and C=O was significantly strengthened. Meanwhile, the vibration strength of C−O and C−H was slightly weakened. The relatively small absorption at 566 cm^−1^ was observed, which was attributed to the Metal–O (M–O) stretching vibrations [33]. The above results confirmed that Fe and Mg affected the pyrolysis process of organic components in rape straw. Previous studies found that the existence of carboxyl groups and hydroxyl groups was conducive to the adsorption of organic contaminant [34,35]. 

**Figure 3:**
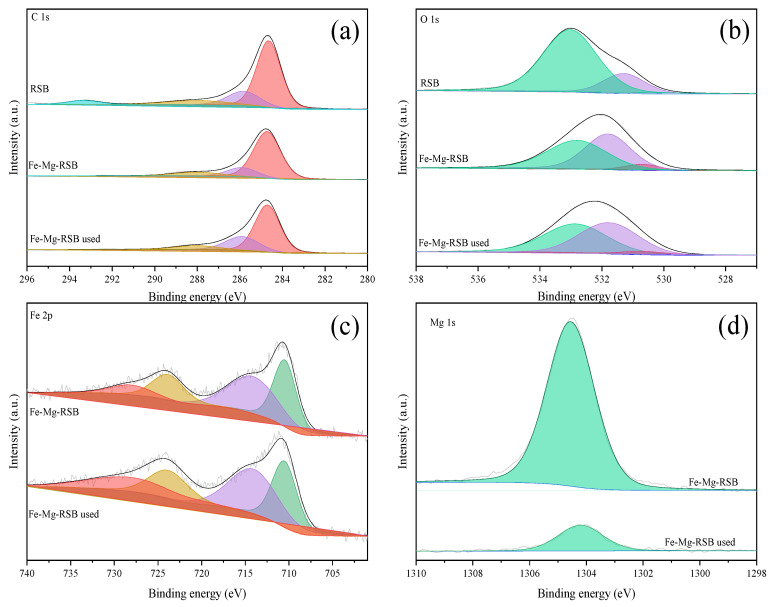
C 1s XPS spectra (**a**). O 1s XPS spectra (**b**). Fe 2p XPS spectra (**c**) and Mg 1s XPS spectra (**d**) of biochars.

The chemical compositions and states of RSB and Fe/Mg-RSB were further analyzed by XPS. Obviously, RSB had the C 1s (284.8 eV) and O 1s (533.0 eV) characteristic peaks (Figure 2d). While after Fe and Mg co-modification, two new characteristic peaks at 711.0 eV (Fe 2p) and 1304.5 eV (Mg 1s) appeared on Fe/Mg-RSB. The C 1s spectrum of RSB was divided into four peaks at 284.7 eV, 285.9 eV, 288.0 eV, and 293.4 eV, corresponding to C−C/C=C, C−O, C=O, and O−C=O, respectively [36] (Figure 3a). However, the peak of O−C=O disappeared after modification. Generally, O−C=O bonds could be converted to C−C/C=C bonds [36]. This indicated that Fe and Mg affected the transformation of C-corresponding bonds. The O 1s spectrum of RSB had been split into two peaks at 532.8 eV and 531.8 eV, belonging to C−O and C=O, respectively [31] (Figure 3b), while C−O decreased and C=O increased by the modification of Fe and Mg, illustrating that the chemical state of O 1s was transformed. A new peak at 530.7 eV was formed on modified biochar, which was attributed to M–O in the metallic oxides [26]. For the Fe 2p spectrum, the peaks at 710.5 eV and 723.8 eV were indexed to Fe 2p3/2 and Fe 2p1/2 of Fe (III), while the other two peaks at 714.1 eV and 728.1 eV could be assigned to Fe 2p3/2 and Fe 2p1/2 of Fe (II) [37] (Figure 3c), which confirmed the formation of Fe (III) and Fe (II) complexes. The above changes resulting from Fe and Mg may further affect the adsorption of organic contaminants.

### 2.2. Batch Adsorption Experiments

#### 2.2.1. Adsorption Kinetics 

**Figure 4:**
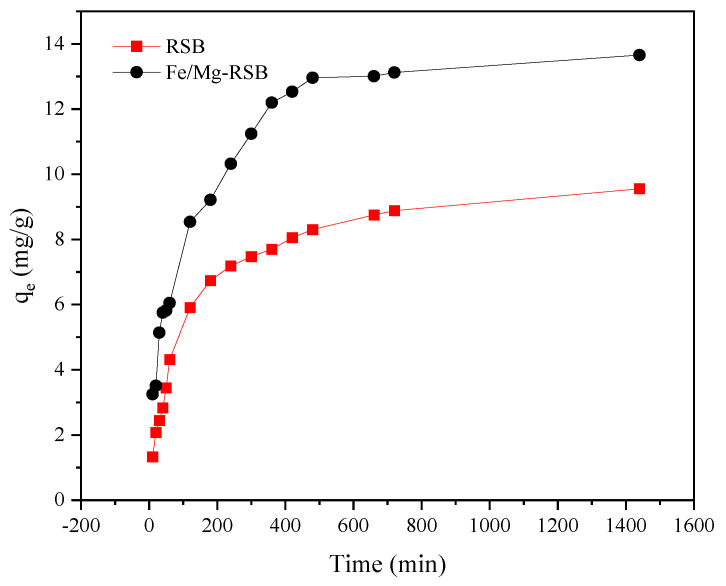
Effect of contact time on the adsorption amount of CQP onto RSB and Fe/Mg-RSB with CQP initial concentration of 10 mg/L.

Figure 4 shows the effect of contact time on the adsorption amount of CQP onto RSB and Fe/Mg-RSB. The adsorption of CQP by RSB and Fe/Mg-RSB increased rapidly in the first 10 min and then gradually descended until reaching adsorption equilibrium after approximately 1440 min. Fe/Mg-RSB showed faster and higher adsorption toward CQP compared with RSB. The removal efficiency of CQP by RSB and Fe/Mg-RSB was 47.76% and 68.30%, respectively. The above results suggested that Fe and Mg co-modification promoted the adsorption of CQP on RSB, which might be owing to its high surface area, developed pore structure, abundant functional groups, and metal ion loading. 

**Figure 5:**
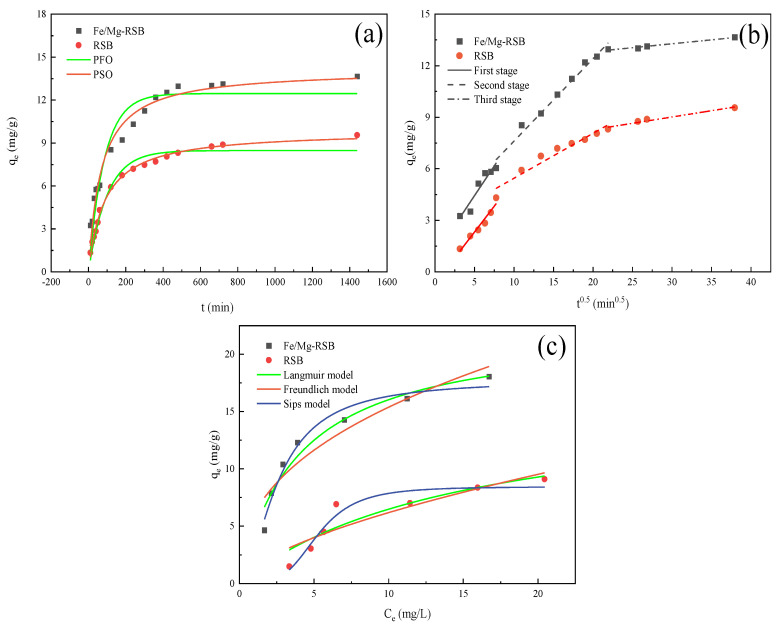
Adsorption kinetics (**a**,**b**) and isotherm (**c**) of CQP on biochars.

Three kinetic models (PFO, PSO, and intra-particle diffusion) were used to fit the above experimental data. The adsorption kinetics of CQP by biochars are displayed in Figure 5a,b. The related adsorption kinetic parameters are shown in Table 1. Compared with the PFO model, the PSO model was more suitable to describe the adsorption behavior, owing to the fact that R^2^ for PSO was higher than that for PFO. Thus, it could be speculated that the adsorption of CQP by biochar was mainly dominated by chemisorption through electron sharing or transfer [38]. According to the PSO model, the qe of CQP on RSB and Fe/Mg-RSB were 9.86 and 14.17 mg/g, which were close to the experimental values of 9.55 and 13.66 mg/g, respectively. In order to further interpret the diffusion behavior, intra-particle diffusion was adopted. As shown in Figure 5b, the adsorption process was split into three linear segments, including film diffusion, intra-particle diffusion, and adsorptive equilibrium phase. The above three linear segments did not pass through the origin, implying that intra-particle diffusion was not the only reason for limiting the speed of the adsorption process and that multiple steps might participate in the adsorption process of CQP onto biochars [39]. It is worth noting that film diffusion and intra-particle diffusion of CQP onto Fe/Mg-RSB processed faster than that onto RSB (Table 1), which indicated that Fe and Mg co-modification enhanced the adsorption rate of RSB for CQP.

#### 2.2.2. Adsorption Isotherms

The experiment data were fitted by three isotherm models (Langmuir, Freundlich, and Sips) (Figure 5c), and adsorption isotherms parameters were shown in Table S2. Langmuir model describes monolayer adsorption behaviors, for example, hydrogen-bonding interaction and precipitation, while the Freundlich model involves multi-layer adsorption behaviors such as electrostatic attraction and van der Waals adsorption, etc. [40]. Sips model is an improved version of the Langmuir and Freundlich models [41]. Based on the correlation coefficient, the Langmuir model better revealed the adsorption behavior of CQP onto biochars, suggesting that monolayer adsorption was predominant. Furthermore, the Sips model possessed the highest R^2^ value among the three isotherm models. The results confirmed that the adsorption of CQP onto biochars was complicated, which might involve not only monolayer adsorption but also a multi-layer adsorption [31]. 

The values of 1/n ranged between 0 and 1, indicating that the adsorption processes were preferable. Meanwhile, the K_F_ of Fe/Mg-RSB was greater than that of RSB, showing that Fe/Mg-RSB had a higher affinity for CQP. Noting that on the basis of the Langmuir model, the maximum adsorption capacity of Fe/Mg-RSB for CQP was 42.93 mg/g (at 308 K), which was about two times higher than that of RSB. Moreover, it was much higher than the adsorption capacity of agar–graphene oxide hydrogel (31 mg/g) [16]. The above results suggested that Fe/Mg-RSB could serve as a potential adsorbent for CQP removal from contaminated water. 

### 2.3. Effects of Reaction Conditions 

#### 2.3.1. Effect of Solution pH

**Figure 6:**
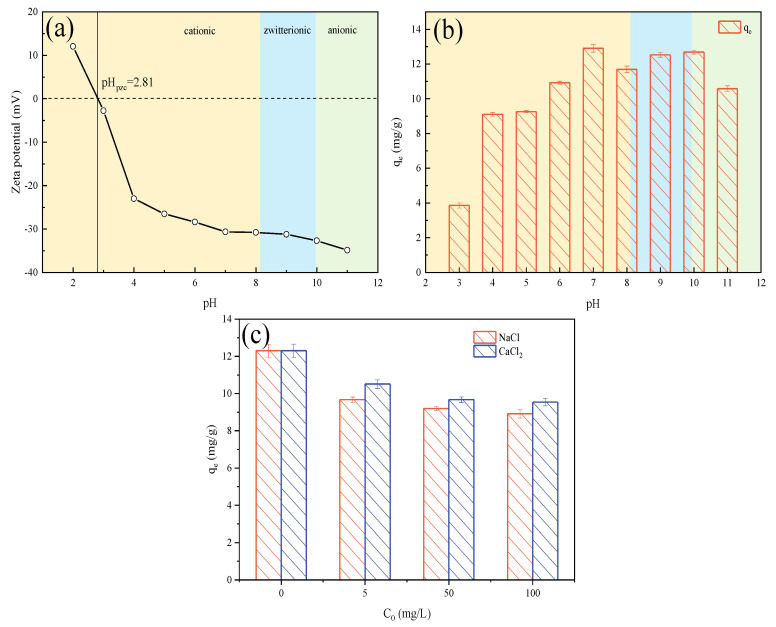
Zeta potential of Fe/Mg-RSB (**a**). Effects of pH (**b**) and ions (**c**) on CQP adsorption by Fe/Mg-RSB.

Solution pH could affect the adsorption performance by varying the ionic species of adsorbate and the surface charge of the adsorbent [42]. CQP has two dissociation constants, pK_a1_ of 8.10 and pK_a2_ of 9.94 [43]. Thus, CQP could present in cationic (pH < 8.10), zwitterionic (8.10 < pH < 9.94), and anionic form (pH > 9.94). The zero charge (pHpzc) of Fe/Mg-RSB was 2.81 (Figure 6a), meaning that the surface of Fe/Mg-RSB was positively charged when pH < pHpzc and conversely, it was negatively charged when pH > pHpzc. 

The adsorption performance of CQP onto Fe/Mg-RSB was investigated under various pH conditions (ranging from 3 to 11) (Figure 6b). The results showed that the CQP adsorption capacity by Fe/Mg-RSB gradually increased with increasing solution pH from 3 to 7, which might be due to the existence of electrostatic interaction between negatively charged Fe/Mg-RSB and cationic CQP. It is noteworthy that the maximum CQP adsorption capacity was reached at pH = 7. However, the CQP adsorption capacity presented a fluctuating decrease trend with further increasing solution pH (8–11). Since Fe/Mg-RSB had a relatively stable adsorption capacity for zwitterionic CQP, the decreased CQP adsorption capacity at pH > 10 might have resulted from the electrostatic repulsion between negatively charged Fe/Mg-RSB and anionic CQP. Notably, the CQP adsorption capacity was still high at a pH of 11, suggesting that electrostatic action was not the only force involved in CQP adsorption.

#### 2.3.2. Effect of Ionic Strength

The effects of ionic strength (NaCl (0–100 mg/L) and CaCl_2_ (0–100 mg/L)) on the CQP adsorption capacity by Fe/Mg-RSB were investigated (Figure 6c). Results showed that the adsorption capacity decreased as the ion concentration increased from 0 to 5 mg/L. It might be because cations (Na^+^ and Ca^2+^) could compete with CQP for active sites on Fe/Mg-RSB under neutral condition [44]. Moreover, the increase in ionic strength reduced the electrostatic attraction between CQP and Fe/Mg-RSB owing to the “screening-out effect” [45]. However, the adsorption capacity only presented a slight decrease when the ion concentration further increased (5 to 100 mg/L), which could be ascribed to the “salting-out effect” [46]. Due to the “salting-out effect”, the solubility of CQP was reduced, and the diffusion of CQP onto Fe/Mg-RSB was promoted, thus preventing the further reduction of the adsorption capacity. Furthermore, the inhibition of the adsorption capacity by Ca^2+^ was lower than that by Na^+^ under the same ion concentration, resulting from the fact that the “salting-out effect” for Ca^2+^ was higher than that for Na^+^ [47]. Overall, Fe/Mg-RSB had stable adsorption performance for CQP in different ionic types and strengths. 

### 2.4. Adsorption Mechanism

The adsorption mechanism of CQP onto Fe/Mg-RSB was implied by the characteristics of adsorbate and adsorbent based on the above analysis. 

First, the surface area and pore volume of biochar played a key role in CQP removal. The BET analysis revealed that Fe/Mg-RSB had a larger surface area and pore volume compared with RSB, suggesting that Fe and Mg co-modification had a good pore-filling effect [39], which could contribute to the improvement of the CQP adsorption capacity. 

In addition to pore filling, the functional groups on biochar also play a crucial role in CQP adsorption. By comparing the positions and intensities of functional groups of Fe/Mg RSB by FTIR before and after CQP adsorption, the underlying roles of such groups were revealed. Firstly, the intensities of aromatic C=C (1615 cm^−1^) and C−O groups (1100 cm^−1^) were strengthened (Figure 2c), suggesting that π-π interaction between Fe/Mg-RSB and CQP facilitated the adsorption process [48,49]. The increase in the intensity of the aromatic C−H group (795 cm^−1^) after adsorption indicated that hydrogen bonding had occurred between Fe/Mg-RSB and CQP. Comparatively, the intensity of M–O (566 cm^−1^) decreased, which proved that CQP could be captured by M–O on the surface of Fe/Mg-RSB through a complexation [31]. 

XPS analysis of Fe/Mg-RSB after CQP adsorption was also performed (Figure 2d and Figure 3). The C1s spectrum showed that the intensities of C−C/C=C (284.7 eV) and C−O (285.9 eV) were enhanced. Additionally, the O 1s spectrum revealed that the peak of C−O (532.8 eV) was intensified. These results further confirmed that π-π interaction between Fe/Mg-RSB and CQP had participated in the adsorption process. It was clear that the content of M–O (530.7 eV) was decreased (Figure 2d). Especially the type and content of Fe changed insignificantly before and after CQP adsorption (Figure 3c). In contrast, the peak intensity of Mg decreased obviously after CQP adsorption (Figure 3d). The above phenomenon indicated that Mg, rather than Fe, was the primary contributor to surface complexation. 

Furthermore, based on the analysis of the influence of pH on adsorption performance, it could be confirmed that electrostatic interaction had a certain promoting effect on CQP adsorption onto Fe/Mg-RSB. 

**Figure 7:**
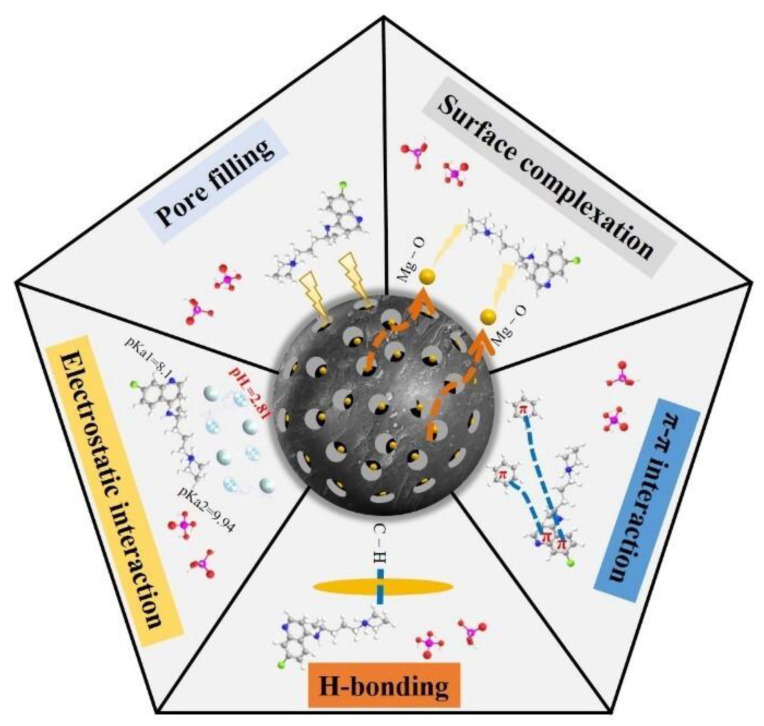
Schematic diagram of adsorption mechanism of CQP adsorption by Fe/Mg-RSB.

In summary, the enhanced adsorption of CQP onto Fe/Mg-RSB was attributed to the synergistic effect of pore filling, π-π interaction, hydrogen bonding, surface complexation, and electrostatic interaction (Figure 7).

### 2.5. Fixed Bed Adsorption of CQP 

**Figure 8:**
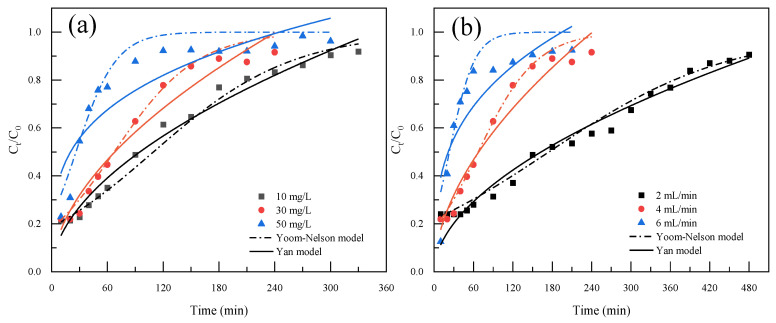
Effects of initial concentration (**a**) (Q_0_ = 4 mg/L, m_0_ = 0.6 g) and inflow rate (**b**) (C_0_ = 30 mg/L, m_0_ = 0.6 g) on breakthrough curves of CQP onto Fe/Mg-RSB. Data were simulated with the Yoom–Nelson model and Yan model.

The effects of initial CQP concentration (10, 30, and 50 mg/L) on adsorption in a fixed bed column were investigated. It was revealed that the slope of the breakthrough curve enhanced with the increasing of the initial CQP concentration (Figure 8a), indicating that the fixed bed column was easier to be penetrated and saturated under a higher concentration gradient because of the larger mass transfer driving force or diffusion coefficient [50]. As the initial CQP concentration increased from 10 to 50 mg/L, the saturation time shortened from 300 to 120 min. This might be attributed to the rapid occupation of active sites by CQP at higher concentration [51]. The breakthrough curves under different flow rates (2, 4, and 6 mL/min) are shown in Figure 8b. At higher flow rates, the breakthrough curve became sharper, and the breakthrough time significantly decreased. One possible explanation was that the residence time of CQP in the column reduced at higher flow rates, leading to the shorter interaction time for the diffusion of CQP into pores and for the adsorption of CQP onto active sites [52].

In order to describe the dynamic CQP adsorption behavior by Fe/Mg-RSB, the breakthrough curves were fitted with the Yoon–Nelson and Yan models. Table 2 showed that the R^2^ values of the Yoon–Nelson model were larger than those of the Yan model, implying that the Yoon–Nelson model was more appropriate for the adsorption process of CQP onto Fe/Mg-RSB. The values of K_YN_ from the Yoon–Nelson model increased with the increase of the initial CQP concentration and the flow rate, whereas the values of τ exhibited the opposite trend. This was likely due to the fact that the saturation of the column could be quickly achieved at a higher initial concentration and flow rate.

### 2.6. Reusability

The reusability of adsorbent is of great importance for its practical application. Herein, EtOH was used as a desorption solvent to investigate the reusability of Fe/Mg-RSB (Figure S1). The CQP adsorption capacity by Fe/Mg-RSB gradually declined with increasing cycle numbers. This might be caused by the decrease of adsorption sites and the block of the pore. However, after five EtOH desorption, the adsorption capacity remained at 73.87% of the first adsorption amount, proving the good reusability of Fe/Mg-RSB.

## 3. Materials and Methods

### 3.1. Materials and Reagents

Rape straw was gathered from Nanjing City, Jiangsu Province, China. The collected samples were naturally dried and washed with deionized water. Thereafter, they were dried in an oven (80 °C) and then ground and passed through a sieve (100 mesh).

Chloroquine phosphate (CQP) was obtained from Aladdin Reagent Co., Ltd. (Shanghai, China). Sodium hydroxide (NaOH), hydrochloric acid (HCl), ferric chloride (FeCl_3_·6H_2_O), magnesium chloride (MgCl_2_·6H_2_O), and ethyl alcohol (EtOH) were purchased from Sinopharm Chemical Reagent Co., Ltd. (Shanghai, China). All the reagents were of analytic grade or higher and were used without further purification.

### 3.2. Synthesis and Characterization of Biochars 

The modified biochar was synthesized with a Fe^3+^/Mg^2+^ molar ratio of 0.35:0.75. Briefly, Rape straw (2 g) was mixed with FeCl_3_·6H_2_O (1.17 g) and MgCl_2_·6H_2_O (3.21 g) in deionized water (200 mL) and then thoroughly stirred for 12 h at room temperature (25 ± 0.5 °C). After filtering, the deposits were placed in the oven for drying at 80 °C. The sample obtained was heated in a tubular furnace under 400 °C for 2 h (rate of 10 °C/min) with continuous nitrogen flow. After natural cooling to room temperature, the product was rinsed with deionized water and dried at 80 °C and was denoted as Fe/Mg-RSB. Pristine rape straw biochar (RSB) was prepared under the same pyrolysis temperature and was used as a control. The characterization methods of the prepared biochars were described in Text S1 of Supplementary Material.

### 3.3. Batch Adsorption Experiments and Data Analysis

The batch adsorption experiments were carried out in 250 mL conical flasks. To investigate adsorption kinetic, 0.05 g Fe/Mg-RSB and 100 mL CQP (10 mg/L) solution were added into a conical flask. The solution was then continuously stirred at the speed of 180 r/min, and the sample was gathered at a certain time. The adsorption isotherms were performed through the mixing of 0.05g Fe/Mg-RSB and 100 mL CQP solution (initial concentration, 4–25 mg/L) at 180 r/min for 8 h with the temperature of 25, 35, and 45 °C. In addition, the effects of reaction conditions on CQP adsorption were investigated by adjusting the following parameters: initial solution pH (3–11) with 0.1 M NaOH or HCL solution, Na^+^ concentration (0–100 mg/L), and Ca^2+^ concentration (0–100 mg/L). The reusability of Fe/Mg-RSB was detected five times using EtOH as a desorption agent.

To minimize error, all experiments were conducted three times. For the analysis of CQP, the supernatant was firstly separated with 0.22 μm syringe filter membrane and then was detected with a UV-vis spectrophotometer (Orion AquaMate 8000, Thermo Scientific, Waltham, MA, USA) at the wavelength of 221 nm. The detection limit of CPQ for UV was 0.26 mg/L. The detailed calculation methods of the adsorption capacity and removal efficiency, as well as the adsorption kinetic model (pseudo-first-order, pseudo-second-order, and intra-particle diffusion model) and isotherm model (Langmuir, Freundlich, and Sips models), were presented in Text S2 of Supplementary Material.

### 3.4. Fixed Bed Column Experiments

The dynamic adsorption behavior was also evaluated in an adsorption column (diameter of 10 mm and height of 200 mm). Typically, 0.6 g Fe/Mg-RSB was packed into the column, which corresponded to a bed height of 4.1 cm. The CQP solution was then added into the column using a peristaltic pump with different initial CQP concentrations (10, 30, and 50 mg/L) and flow rates (2, 4, and 6 mL/min). The flow rate was controlled by the peristaltic pump. The column effluent was collected at regular time intervals, and the concentration of CQP was measured by the UV-vis spectrophotometer. In order to assess the adsorption performance in the column, the experimental data were analyzed with two models (Text S3 in Supplementary Material).

## 4. Conclusions

Herein, a well-performance biochar (Fe/Mg-RSB) was successfully prepared. Compared with RSB, Fe/Mg-RSB had a larger surface area, a more developed pore structure, and functional groups. These characteristics collectively contributed to significantly enhancing the adsorption capacity of Fe/Mg-RSB towards CQP. The maximum adsorption capacity of Fe/Mg-RSB for CQP could reach 42.93 mg/g (at 308 K). The adsorption mechanism involved pore filling, π-π interaction, hydrogen bonding, surface complexation, and electrostatic interaction.

## Figures and Tables

**Table 1:** Adsorption kinetic parameters for CQP on biochars.

Kinetic Equation	Parameters	RSB	Fe/Mg-RSB
Pseudo-first-order	q_m_ (mg/g)	8.47	12.46
	K_1_ (min^−1^)	0.01	0.01
	R^2^	0.97	0.919
Pseudo-second-order	q_m_ (mg/g)	9.86	14.17
	K_2_ (g/(mg·min))	11.06	41.74
	R^2^	0.995	0.971
Intra-particle diffusion	K_1_ (mg/(g·min^0.5^))	0.601	0.691
	R^2^	0.952	0.907
	K_2_ (mg/(g·min^0.5^))	0.261	0.479
	R^2^	0.941	0.982
	K_3_ (mg/(g·min^0.5^))	0.073	0.045
	R^2^	0.964	0.971

**Table 2:** Fitting results of Yoon–Nelson and Yan dynamic adsorption model.

Operation Parameters		m_0_ (g)	Yoon-Nelson Model			Yan Model		
C_0_ (mg/L)	Q_0_ (mL/min)		K_YN_ (min^−1^)	τ (min)	R^2^	q_y_ (mg/g)	a	R^2^
10	4	0.6	0.0134	109.465	0.985	8.364	0.533	0.978
30	4	0.6	0.0225	67.166	0.979	17.381	0.547	0.954
50	4	0.6	0.0466	26.166	0.918	29.419	0.278	0.820
30	2	0.6	0.0073	174.907	0.985	21.409	0.536	0.964
30	6	0.6	0.0524	23.277	0.839	21.057	0.314	0.780

## Data Availability

Data is contained within the article or the supplementary data file.

## Supplementary Materials

The following supporting information can be downloaded at: https://www.mdpi.com/article/10.3390/molecules28083290/s1, Text S1: Characterization of adsorbents. Text S2: Experimental data analysis. Text S3: Fixed bed column data analysis. Figure S1: Reusability experiment of Fe/Mg-RSB. Table S1: Pore characterization parameters of RSB and Fe/Mg-RSB. Table S2: Parameters of isotherm models for CQP adsorption on RSB and Fe/Mg-RSB.
