# Supplementary material for: Removal Performance and Mechanism of Emerging Pollutant Chloroquine Phosphate from Water by Iron and Magnesium Co-Modified Rape Straw Biochar
Source: Molecules. 2023 Apr 7;28(8):3290. doi: 10.3390/molecules28083290 (PMC10146006; doi:10.3390/molecules28083290)
Supplement: Supplementary file 1 [file molecules-28-03290-s001.zip › molecules-2259982-supplementary.pdf]

Removal Performance and Mechanism of Emerging Pollutant Chloroquine Phosphate from Water by Iron and Magnesium Co-Modified Rape Straw Biochar

Hongwei Sun [1], Jinjin He [1], Yucan Liu [2], Xianguo Ji [2], Gang Wang [1], Xiaoyong Yang [1] and Yanxiang Zhang [1,*]

[1] School of Environmental and Materials Engineering, Yantai University, Yantai 264005, China
[2] School of Civil Engineering, Yantai University, Yantai 264005, China
* Correspondence: 15092166641@163.com or yanxiangzhang@ytu.edu.cn; Tel.: +86-156-9961-3180

Text S1. Characterization of adsorbents

The surface morphology of biochar was analyzed with a Scanning Electron Microscope-Energy Dispersive Spectrometer (SEM-EDS, Carl Zeiss AG, Sigma 500, Germany). Fourier Transform Infrared Spectroscopy (FTIR, Thermo Scientific, Nicolet iS5, USA) was used to determine the functional groups of biochar. The surface area and pore size were characterized by Brunauer-Emmett-Teller (BET, Micromeritics ASAP 2460, USA). The chemical state and surface chemical composition of biochar before and after removal of CQP were analyzed by X-ray photoelectron spectroscopy (XPS, Thermo Scientific Escalab 250Xi, USA). The zeta potential of Fe/Mg-RSB was obtained by Malvern Zetasizer Nano (ZEN3700).

Text S2. Experimental data analysis

The adsorption capacity q_e (mg/g) and removal efficiency R (%) were used to evaluate the adsorbent (Eqs. (S1, S2)):

$$q_e = \frac{(C_0\text{-}C_e) \times V}{m} \quad \text{(S1)}$$

$$R = \frac{C_0\text{-}C_e}{C_0} \times 100\% \quad \text{(S2)}$$

Where C_0 (mg/L) and C_e (mg/L) are initial and equilibrium CQP concentrations, respectively; V (L) is the volume of solution; m (g) is the adsorbent dosage.

The pseudo first order (PFO-order), pseudo second order (PSO-order), and intra-particle diffusion models were utilized to fit the adsorption kinetics (Eqs. (S3)-(S5)):

$$q_t = q_e(1\text{-}\exp(\text{-}K_1 t)) \quad \text{(S3)}$$

$$q_t = \frac{K_2 q_e^2 t}{1+k_2 q_e t} \quad \text{(S4)}$$

$$q_e = Kt^{0.5} + C \quad \text{(S5)}$$

Where q_e (mg/g) and q_t (mg/g) are the adsorption capacity of the adsorbent to

CQP under equilibrium and time t (min), respectively; K_1 (min^{-1}) and K_2 (g/(mg·min)) are PFO and PSO rate constants, respectively; K (mg/(g·$min^{0.5}$)) is the intra-particle diffusion rate constant; C (mg/g) is the intra-particle diffusion constant.

Three isotherm models (Langmuir, Freundlich and Sips) were selected to describe the adsorption isotherm data (Eqs. (S6)-(S8)):

$$q_e = \frac{q_m K_L C_e}{1+K_L C_e} \quad (S6)$$

$$q_e = K_F C_e^{1/n} \quad (S7)$$

$$q_e = \frac{q_m (K_S C_e)^m}{1+(K_S C_e)^m} \quad (S8)$$

Where C_e (mg/L) is the equilibrium concentration; q_e (mg/g) and q_m (mg/g) are the equilibrium and maximum adsorption capacity, respectively; K_L (L/mg) is the constant of Langmuir model; K_F ($L^{1/n}$·$mg^{1-1/n}$·g^{-1}) and n are Freundlich model constants; K_S (L/mg) and m are Sips model constants.

Text S3. Fixed bed column data analysis

The Yoom-Nelson model and Yan model were used to study the kinetics of column adsorption (Eqs. (S9, S10)):

$$\ln\left(\frac{C_e}{C_0-C_e}\right) = K_{YN}t - K_{YN}\tau \quad (S9)$$

$$\frac{C_e}{C_0} = 1 - \frac{1}{1+\left(\frac{C_0 Qt}{q_y m}\right)^{\alpha}} \quad (S10)$$

Where C_e (mg/L) and C_0 (mg/L) are effluent and influent concentration, respectively; K_{YN} (min^{-1}) and α are Yoom-Nelson and Yan model constants; τ (min) is the time required for retaining 50% of the initial adsorbate breakthrough; q_y (mg/g) is the maximum adsorption capacity; m (g) is the

dosage of adsorbent in the column; Q (mL/min) is the flow rate; t (min) is the adsorption time.

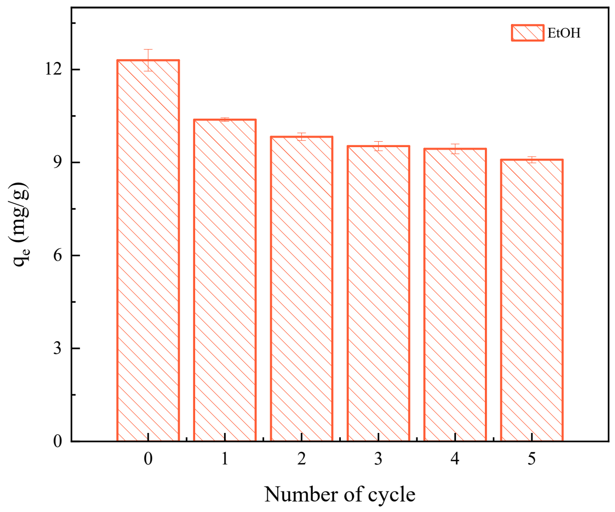

Fig. S1. Reusability experiment of Fe/Mg-RSB.

Table S1 Pore characterization parameters of RSB and Fe/Mg-RSB.

Biochars	BET surface areas (m^2/g)	Pore volume (cm^3/g)	Pore size (nm)	Particle size (μm)
RSB	20.7053	0.0189	3.6488	91.20
Fe/Mg-RSB	25.3346	0.0231	3.6326	79.43

Table S2 Parameters of isotherm models for CQP adsorption on RSB and Fe/Mg-RSB.

Adsorbent	Temperature (K)	Langmuir			Freundlich			Sips			
		q_m (mg/g)	K_L (L/mg)	R^2	K_F ($L^{1/n}\cdot mg^{1-1/n}\cdot g^{-1}$)	1/n	R^2	q_m (mg/g)	K_S (L/mg)	m	R^2
RSB	298 K	16.36	0.06	0.853	1.47	0.62	0.821	8.44	0.19	4.02	0.937
	308 K	20.79	0.09	0.938	2.43	0.58	0.893	13.13	0.19	2.05	0.981
	318 K	22.19	0.12	0.943	3.40	0.53	0.899	15.01	0.24	2.07	0.981
Fe/Mg-RSB	298 K	22.38	0.25	0.951	6.07	0.43	0.894	17.77	0.39	1.80	0.974
	308 K	42.93	0.09	0.886	4.43	0.65	0.873	29.57	0.18	1.36	0.890
	318 K	54.62	0.08	0.915	4.70	0.70	0.915	97.38	0.03	0.84	0.916
